# Supplementary material for: Smooth Interpolating Curves with Local Control and Monotone Alternating Curvature
Source: Comput Graph Forum. 2022 Oct 6;41(5):25–38. doi: 10.1111/cgf.14600 (PMC9827861; doi:10.1111/cgf.14600)
Supplement: Supplementary file 1 — Supplement Material [file CGF-41-25-s001.zip › Local-Smooth-Interpolating-MonoCurvature/extern/clothoids/docs/api-matlab/class_a00088.html]

Class ClothoidSplineG2 — Clothoids v2.0.9

### Navigation

- index
- toc
- next
- previous
- Clothoids »
- MATLAB API »
- Class ClothoidSplineG2

# Class ClothoidSplineG2¶

- Defined in File ClothoidSplineG2.m

## Inheritance Relationships¶

### Base Type¶

- `public handle`

## Class Documentation¶

class ClothoidSplineG2 : public handle¶
:   Construct a piecewise clothoids \( \G(s) \) composed by n clothoids that solve the G2 problem

    **match points**

    \[ G(s\_k) = \mathbf{p}\_k \]

    \[ \lim\_{s\to s\_k^+} G'(s) = \lim\_{s\to s\_k^-} G'(s) \]

    \[ \lim\_{s\to s\_k^+} G''(s) = \lim\_{s\to s\_k^-} G''(s) \]

    **Reference**

    The solution algorithm is described in

    - **E.Bertolazzi, M.Frego**, Interpolating clothoid splines with curvature continuity Mathematica Methods in the Applied Sciences, vol 41, N.4, pp. 1723-1737, 2018

    Public Functions

    function nlsys( self, theta)¶

    function con( self, theta)¶

    function obj( self, theta)¶

    function build( self, x, y)¶
    :   Call C++ setup for the problem solver. Check that consecutive points are distinct

    function build\_internal( self, x, y, varargin)¶

    function build\_internal2( self, x, y, varargin)¶

    function ClothoidSplineG2()¶

    function verbose( self, yes)¶

    function ipopt( self, yesno)¶

    function ipopt\_check( self, yesno)¶

    function dims( self)¶

    function guess( self)¶

    function buildP1( self, x, y, theta0, theta1)¶
    :   Compute the clothoid spline passing to the points (x(i),y(i)) with initial angle theta0 (radiants) and final angle theta1 (radiants)

    function buildP2( self, x, y)¶
    :   Compute the clothoid spline passing to the points (x(i),y(i)) with cyclic condition (tangent and curvature meet)

        - theta(1) = theta(end) mod 2\*pi
        - kappa(1) = kappa(end)

    function buildP3( self, x, y, theta0, kappa0)¶
    :   Compute the clothoid spline passing to the points (x(i),y(i)) with assignede initial angle and survature NB: this target is not reccomended (its unstable), used only for debugging

    function buildP4( self, x, y)¶
    :   Compute the clothoid spline passing to the points \( (x\_i,y\_i)\) and minimizing derivative of the curvature al the extrema

        minimize \( k'(0)^2 + k'(L)^2 \)

    function buildP5( self, x, y)¶
    :   Compute the clothoid spline passing to the points \( (x\_i,y\_i)\) and minimizing the length of the first and last segment

        minimize \( (s\_1-s\_0)+(s\_n - s\_{n-1}) \)

    function buildP6( self, x, y)¶
    :   Compute the clothoid spline passing to the points \( (x\_i,y\_i)\) and minimizing the total length of the spline

        \( L = s\_n-s\_0 \)

    function buildP7( self, x, y)¶
    :   Compute the clothoid spline passing to the points \( (x\_i,y\_i)\) and minimizing the integral of the square of the curvature:

        minimize: \( \displaystyle \int\_0^L k(s)^2 \mathrm{d}s \)

    function buildP8( self, x, y)¶
    :   Compute the clothoid spline passing to the points \( (x\_i,y\_i)\) and minimizing the integral of the square of the curvature derivative:

        minimize: \( \displaystyle \int\_0^L k'(s)^2 \mathrm{d}s \)

    function buildP9( self, x, y)¶
    :   Compute the clothoid spline passing to the points \( (x\_i,y\_i)\) and minimizing the integral of the jerk squared

### Quick search

### Table of Contents

- Matlab Interface Manual
- C++ API
- MATLAB API

«
hide menu

menu
sidebar
»

### Navigation

- index
- toc
- next
- previous
- Clothoids »
- MATLAB API »
- Class ClothoidSplineG2

© Copyright 2021, Enrico Bertolazzi and Marco Frego.
Created using Sphinx 4.2.0.
